# Increased Expression and Activity of Brain Cortical cPLA2 Due to Chronic Lipopolysaccharide Administration in Mouse Model of Familial Alzheimer’s Disease

**DOI:** 10.3390/pharmaceutics14112438

**Published:** 2022-11-10

**Authors:** Mikko Gynther, Mariana Leal Estrada, Sanna Loppi, Paula Korhonen, Katja M. Kanninen, Tarja Malm, Jari Koistinaho, Seppo Auriola, Gert Fricker, Elena Puris

**Affiliations:** 1 Institute of Pharmacy and Molecular Biotechnology, Ruprecht-Karls-University, Im Neuenheimer Feld 329, 69120 Heidelberg, Germany; 2 A.I. Virtanen Institute for Molecular Sciences, University of Eastern Finland, P.O. Box 1627, 70211 Kuopio, Finland; 3 Department of Immunobiology, University of Arizona, 1656 E Mabel Street, Tucson, AZ 85724-5221, USA; 4 Neuroscience Center, Helsinki Institute for Life Science, University of Helsinki, Haartmaninkatu 8, 00290 Helsinki, Finland; 5 School of Pharmacy, University of Eastern Finland, P.O. Box 1627, 70211 Kuopio, Finland

**Keywords:** Alzheimer’s disease, inflammation, cytosolic phospholipase A2, LPS, mice

## Abstract

Cytosolic phospholipase A2 (cPLA2) is an enzyme regulating membrane phospholipid homeostasis and the release of arachidonic acid utilized in inflammatory responses. It represents an attractive target for the treatment of Alzheimer’s disease (AD). Previously, we showed that lipopolysaccharide (LPS)-induced systemic inflammation caused abnormal lipid metabolism in the brain of a transgenic AD mouse model (APdE9), which might be associated with potential changes in cPLA2 activity. Here, we investigated changes in cPLA2 expression and activity, as well as the molecular mechanisms underlying these alterations due to chronic LPS administration in the cerebral cortex of female APdE9 mice as compared to saline- and LPS-treated female wild-type mice and saline-treated APdE9 mice. The study revealed the significant effects of genotype LPS treatment on cortical cPLA2 protein expression and activity in APdE9 mice. LPS treatment resulted in nuclear factor kappa-light-chain-enhancer of activated B cells (NFkB) activation in the cortex of APdE9 mice. The gene expressions of inflammation markers Il1b and Tnfa were significantly elevated in the cortex of both APdE9 groups compared to the wild-type groups. The study provides evidence of the elevated expression and activity of cPLA2 in the brain cortex of APdE9 mice after chronic LPS treatment, which could be associated with NFkB activation.

## 1. Introduction

Phospholipase A2 (PLA2) mediates the cleavage of acyl groups at the sn-2 positions of substrate phospholipids in the cell membrane resulting in the production of a free fatty acid and a lysophospholipid [1]. Investigating PLA2s has been of interest to researchers, as substrates of these enzymes play important roles in regulating cell immune functions and inflammatory responses [1,2]. The superfamily of PLA2s consists of more than 50 subtypes of enzymes based on their cellular localization and calcium dependence [2]. One group of the PLA2s which has been considered as an attractive drug target for the treatment of neurodegenerative diseases, including Alzheimer’s disease (AD), is calcium-dependent cytosolic phospholipases A2 (cPLA2s) [3].

Group IV PLA2α (cPLA2α), which is ubiquitously expressed in all brain cells, including neurons, astrocytes, and microglia, is the most studied among cPLA2s [4]. This enzyme mediates the release of arachidonic acid and lysophosphatidylcholine (LPC) from the brain membrane phosphatidylcholine (PC) [3]. The activation and translocation of the cPLA2α enzyme to membranes occurs after an increase in the concentration of cytosolic calcium due to the production of diacylglycerol (DAG) and inositol triphosphate (IP3) [5,6]. The activation of cPLA2α occurs via the phosphorylation of the enzyme at several serine residues by the mitogen-activated protein kinase (MAPK) [5,6]. cPLA2α plays a significant role in neurotransmission by acting as a signaling molecule [7,8,9]. In addition, cPLA2α has an important function in neuroinflammation via providing precursors to produce different eicosanoids by cyclooxygenases, lipoxygenases, and prostaglandin synthases [7,8,9]. Moreover, activation of cPLA2α boosted by sphingolipid metabolism promoted nuclear factor kappa-light-chain-enhancer of activated B cells (NF-κB) activation via interaction with mitochondrial antiviral-signaling protein (MAVS) in astrocytes [10]. In addition, Chuang et al. (2015) demonstrated that the enzyme plays an important role in reactive oxygen species (ROS) and nitric oxide (NO) signaling during microglial activation [11].

Multiple studies have focused on identifying the role of cPLA2α in the onset of AD and they have shown that cPLA2 might be a key player in AD pathogenesis [12]. The first evidence of the contribution of cPLA2α in AD was demonstrated by elevated levels of the enzyme immunoreactivity in AD brain [13]. Moreover, different studies indicated the involvement of cPLA2α in Aβ-triggered biochemical changes in astrocytes and microglial cells [14,15]. Palavicini et al. (2017) showed that oligomeric Aβ activated cPLA2α through the stimulation of MAPK cascade [16]. Sanchez-Mejia et al. (2008) demonstrated that reduction of cPLA2 can prevent diverse Aβ-mediated functional impairments, such as learning and memory deficits, behavioral alterations, and premature mortality in transgenic mice with neuronal expression of familial AD-mutant human amyloid precursor protein (hAPP) [17].

Systemic inflammation may contribute to the onset of AD. Multiple epidemiological and animal studies have provided evidence that systemic inflammatory conditions caused by peripheral infections may be associated with a greater risk of AD and can accelerate disease progression [18,19]. In APPswe transgenic mice, the systemic administration of an endotoxin lipopolysaccharide (LPS) led to enhanced APP expression and increased intracellular accumulation of Aβ [20]. In addition, wild-type (WT) mice treated systemically with LPS produced pro-inflammatory cytokines, such as interleukin-1 beta (IL-1β) and tumor necrosis factor alpha (TNF-α) in the brain, as result of peripheral stimulation [21]. Interestingly, both pro-inflammatory cytokines have shown activation effects on cPLA2 [22,23]. However, the effect of systemic inflammation on cPLA2 activity and expression in vivo has not been studied.

In our previous study, we investigated the effects of chronic systemic LPS treatment on plasmatic and brain metabolome and lipidome in the APdE9 mouse model of AD [24]. Our study revealed that chronic LPS administration resulted in a greater number of elevated DAGs and decreased PCs in the brain cortical tissue of APdE9 mice compared to saline-treated WT and APdE9 mice and LPS-treated WT mice [24]. Moreover, we observed significantly increased levels of several LPCs in the brain cortical tissue of both saline- and LPS-treated APdE9 mice [24]. The mentioned lipid pathways involve cPLA2α. Therefore, the aim of the present study was to investigate the potential systemic LPS dosing-induced changes in cPLA2α expression and enzymatic activity, as well as the molecular mechanisms underlying these alterations in APdE9 mice as compared to saline-treated WT and APdE9 mice as well as LPS-treated WT mice.

## 2. Materials and Methods

### 2.1. Materials

Acetonitrile, dithiothreitol, urea, ethylenediaminetetraacetic acid (EDTA), guanidine hydrochloride, Tris-HCl, formic acid, LPS (#L2880), and tribromoethanol (#75-80-9, Avertin) were purchased from Sigma-Aldrich (St. Louis, MO, USA). The stable isotope-labelled quantified peptides were purchased from JPT Peptide Technologies GmbH, Berlin, Germany. Tosylphenylalanylchloromethyl ketone-treated trypsin (#VA9000), Protease Max surfactant (#V2072), and lysyl endopeptidase (Lys-C, #VA1170) were purchased from Promega (Madison, WI, USA). BioRad DC Protein Assay was purchased from EnVision (PerkinElmer Inc., Waltham, MA, USA).

### 2.2. Study Design and Animals

The animals and treatment regimen were the same as in our previous study [24]. The animal experiments complied with the ARRIVE guidelines and were conducted according to EU Directive 2010/63/EU for animal experiments. The animal procedures were approved by the Finnish National Animal Experimental Board on 10 March 2015 with the license number ESAVI-2015-000744. Only healthy animals with no signs of illness as examined visually and by body weight were used in the study. The APdE9 transgenic mice (RRID: MGI:5701399) used in the study were generated by co-injection of a chimeric mouse or human APP695 harboring the Swedish mutation and human PS1-dE9 vectors, both controlled by their own mouse prion protein promoter element [25]. The double transgenic mice were backcrossed to the C57BL/6J strain to create APdE9 transgenic (APP/PS1) mice in C57BL/6J background. Wild-type (WT) siblings were used as controls. All of the mice were 16–17 months old, as at this age the APdE9 mice are characterized by elevated formation of Aβ plagues as well as learning and memory deficits [26,27,28]. In the study, only female mice were used due to the higher prevalence of AD among women compared to men [29].

The animals were divided into one of the following study groups as previously described [24]: the WT mice treated chronically with LPS (WT plus LPS group); saline-treated APdE9 mice (APdE9 group), APdE9 mice treated chronically with LPS (APdE9 plus LPS group), or saline-treated WT mice (WT group). The chronic LPS treatment used in the study has been previously used to induce inflammation in APdE9 mice [24]. Briefly, WT and APdE9 mice were administered 500 µg/kg i.p. LPS twice a week for four weeks followed by a two-week wash-out period. After that, 500 µg/kg i.p. LPS were administered to mice twice a week for four weeks. Following LPS administration, a five-week wash-out period was used to ensure that the observed changes were chronic and maintained after termination of LPS treatment. After the wash-out period, the animals were decapitated. The mice in the WT and APdE9 groups were administered 0.9% saline solution i.p. instead of LPS using the same regimen as the animals treated with LPS.

Standard laboratory conditions were used for housing the animals: 12-12 h light-dark day cycle, 60% relative humidity, food (Lactamin R36; Lactamin AB, Södertälje, Sweden), and water consumption ad libitum. There were no statistically significant differences in the body weight of the animals between the study groups: WT group (26 ± 2.0 g), WT plus LPS group (26 ± 1.4 g), APdE9 group (27 ± 1.1 g), APdE9 plus LPS group (27 ± 2.0 g). All experimental procedures were performed during the daytime. On the decapitation day, the animals were anesthetized by tribromoethanol, a terminal anesthetic, which provides fast and deep surgical analgesia. The use of tribromoethanol was approved by the Finnish National Animal Experimental Board (ESAVI-2015-000744). After anesthesia, the mouse brains were perfused using 3-min transcardial perfusion with heparinized saline (2500 IU/L). The meninges were removed, and the brain cortex was collected, snap-frozen in liquid nitrogen, and stored at −80 °C until further analysis.

### 2.3. Quantitative Targeted Absolute Proteomic (QTAP) Analysis

The absolute protein expression levels of cPLA2α were measured in the mouse brain cortical homogenate. The total protein concentrations in the brain cortical homogenate were measured using the BioRad DC Protein Assay. The samples were prepared according to the previously published protocol [30,31]. The aliquots of brain homogenate containing 50 μg of total protein were evaporated under nitrogen stream in a cold-water bath. The samples were solubilized in 9 μL of 100 mM Tris–HCl (pH 8.5) and 6 M urea followed by reduction with dithiothreitol and *S*-carbamoylmethylation with iodoacetamide. The proteins were precipitated with methanol and chloroform, and the precipitates were dissolved in 6 M urea in 0.1 M Tris–HCl (pH 8.5). The samples were diluted five times with 0.1 M Tris–HCl (pH 8.5), which was spiked with an internal standard peptide, which was specific for human and mouse cPLA2α (IYEPLDVK). In the peptide, lysine (K) was labelled by a stable isotope ^13^C and ^15^N. After that, Lys-C and Protease-Max were added, and the samples were incubated at room temperature for 3 h. Tosylphenylalanyl chloromethyl ketone-treated trypsin was added to the samples (enzyme/substrate ratio of 1:100) for tryptic digestion. The samples were incubated at 37 °C for 16 h. To acidify the samples, formic acid in water 20% (*v*/*v*) was added. The samples were centrifugated at 14,000× *g* for 5 min at 4 °C. The supernatants were analyzed using the LC-MS/MS methods described below. 

The LC-MS/MS analysis was performed using an Agilent 1290 Infinity LC (Agilent Technologies, Waldbronn, Germany), which was coupled to an Agilent 6495 Triple Quadrupole Mass Spectrometer equipped with an ESI source (Agilent Technologies, Palo Alto, CA, USA). The HPLC separation and elution of peptides was performed using Advance Bio Peptide Map column (2.1 × 250 mm, 2.7 μm) as described previously [30,31]. The positive ion multiple reaction monitoring (MRM) mode was used for the detection of peptides. The dwell time of 20 ms per transition was used. The ion source temperature was set to 210 °C, while the drying gas flow rate was 16 L/min. The nebulizer pressure was 45 psi. The MS capillary voltage was 3 kV. The quantitation of the target protein was based on one unique peptide (IYEPLDVK) selected according to the in silico criteria for peptide selection [32]. The cPLA2α selective peptides were monitored with four different MRM transitions derived from the stable isotope-labelled peptide (492.7→871.4, 492.7→708.4, 492.7→579.3) and the unlabeled natural peptides (488.7→863.4, 488.7→700.4, 488.7→571.3). The Agilent MassHunter Workstation Acquisition software (Agilent Technologies, Data Acquisition for Triple Quad., version B.03.01, Santa Clara, CA, USA) was used for acquiring the data. The data were processed with Skyline software (version 4.1, MacCoss Lab Software, Seattle, WA, USA). 

### 2.4. Measurement of cPLA2 Activity

The activity of cPLA2 was determined in brain cortical homogenates using the Abcam Cytosolic Phospholipase A2 Assay Kit # ab133090 (Abcam, Cambridge, MA, USA). The mouse brain cortical tissue was homogenized in 5 mL of cold buffer (i.e., 50 mM HEPES (Sigma-Aldrich, St. Louis, MO, USA), pH 7.4, containing 1 mM EDTA) per gram of tissue, followed by centrifugation at 10,000× *g* for 15 min at 4 °C. The supernatant was immediately stored on ice and used for assay according to the manufacturer’s instructions. The absorbance was measured at 414 nm in a Sunrise™ absorbance microplate reader, Tecan (Männedorf, Switzerland).

### 2.5. Quantitative Reverse Transcription Polymerase Chain Reaction (qRT-PCR)

Gene expression of Pla2g4a, pro-inflammatory cytokines interleukin-1 beta (Il1b) and tumor necrosis factor alpha (Tnfa) as well as oxidative stress markers sirtuin-3 (Sirt3) and oxidative stress responsive kinase 1 (Oxsr1) in the brain cortices of investigated mouse models were quantified by qRT-PCR analysis. First, the RNeasy Mini Kit (#74004, Qiagen, Stockach, Germany) was used for extraction of total RNA from the mouse brain cortices according to the manufacturer’s instructions. Total RNA was quantified using NanoDrop (Thermo Scientific, Dreieich, Germany). Consequently, cDNA was synthesized using the Biozym cDNA synthesis Kit (#331475S, Biozym Scientific GmbH, Oldendorf, Germany) according to the manufacturer’s instructions. Total RNA (1 μg) was used as a template for reverse transcription in 20 μL reaction volumes and then diluted with RNase-free water. The synthesized cDNA was mixed with the PowerUp™ SYBR™ Green Master Mix (#A25741, Thermo-Fischer, Waltham, MA, USA) and specific gene primers (Table 1), which were purchased from Thermo-Fisher Scientific (Dreieich, Germany). The primers were designed using Primer-BLAST (National Library of Medicine, Bethesda, MD, USA) and OligoAnalyzer v. 3.1 (Integrated DNA Technologies, Inc., Redwood City, CA, USA) and validated using thermal gradient and gel electrophoresis. Standard curve validation was performed, and amplification efficiency was determined from the slope, and ranged between 90% and 110%. 

Relative target gene expression normalized to gene expression of the housekeeping gene, beta-actin (Actb), in each sample was estimated according to the method explained previously [33]. For qRT-PCR analysis, LightCycler 96 (Roche Diagnostics) was used. The data were acquired using LightCycler^®^ 96 SW 1.1 software, v. 1.1.0.1320 (Roche Diagnostics, Mannheim, Germany; 2011).

### 2.6. NFκB p65 Phosphorylation Analysis

The phosphorylated and total NFκB p65 expression was measured in the brain cortices of the investigated mouse models. The brain cortical tissues of the investigated animals were homogenized in lysis buffer to achieve a final total protein concentration of 0.5 μg total protein/μL. The total protein concentration in the lysates was measured by Bio-Rad Protein Assay (EnVision, PerkinElmer, Inc., Waltham, MA, USA). The phosphorylated and total NFκB p65 expression in the cell lysates was measured by a NFκB p65 (Total/Phospho) Human InstantOne™ ELISA Kit (# 85-86083-11, Invitrogen, Carlsbad, CA, USA) according to the kit instructions.

### 2.7. Measurement of Reactive Oxygen Species (ROS) Production

ROS production was measured in the brain cortical tissues of the investigated mouse models. The brain homogenates were obtained in a similar way to the way they were obtained for the cPLA2 activity measurement and used with a final protein concentration of 0.1 μg total protein/μL. The production of total ROS was measured by the ROS-ID^®^ Total ROS/Superoxide detection kit (ENZ-51010, Enzo Life Sciences, Farmingdale, NY, USA) according to the manufacturer’s instructions. Fluorescence was measured by a TECAN infinite F200 Pro fluorescence plate reader with excitation and emission wavelengths of 540 nm and 590 nm, respectively.

### 2.8. Statistical Analysis

This is an exploratory study. The sample size (*n* = 4–6) used in the present study was based on previous similar studies in mice (*n* = 3–5) [30,34,35]. The data are presented as mean ± standard deviation (SD) for the absolute protein expression levels, normalized mRNA expression. The statistical significance of the differences in normalized fold changes in the mRNA expression of Pla2g4a, Il1b, Tnfa, Sirt3 as well as cPLA2 protein expressions and activities, ROS production and NFκB p65 phosphorylation between the WT plus LPS, APdE9, APdE9 plus LPS groups and the WT group was analyzed by a non-parametric Kruskal–Wallis test followed by a Dunn’s multiple comparisons post-hoc test. A *p*-value < 0.05 was considered statistically significant. Data analysis was completed using GraphPad Prism, version 9.0 (GraphPad Software, San Diego, CA, USA).

## 3. Results

### 3.1. Expression and Activity of cPLA2α

**Figure 1:**
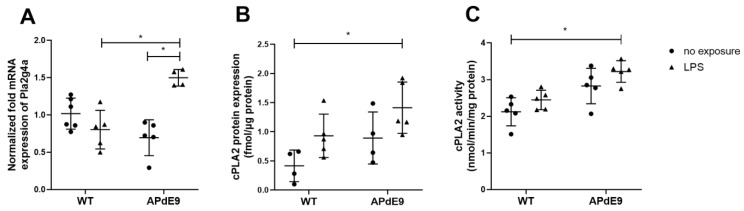
Comparison of normalized fold mRNA expression of Pla2g4a (**A**), absolute protein ex-pression levels of cPLA2α (**B**) and cPLA2 enzyme activity (**C**) in the cerebral cortical tissue in saline-treated female wild-type (WT) control mice vs. female WT mice treated with LPS, and female APdE9 mice treated with either saline or LPS. The line represents group mean and whiskers—the SD (*n* = 4–6 per group). The statistical significance of changes in cPLA2 gene and protein expressions as well as the activity between the groups was analyzed by a Kruskal–Wallis test followed by a Dunn’s multiple comparisons post-hoc test. Statistically significant differences are marked with asterisks, where * indicates *p* < 0.05.

In the present study, we assessed Pla2g4a gene expression changes in the brain cortices of exposed mice (Figure 1A). A Kruskal–Wallis analysis detected a significant difference between the study groups (*p* = 0.005). Therefore, a Dunn’s multiple comparisons post-hoc test was used for comparison of Pla2g4a normalized mRNA expression between the treatment groups. As a result, a statistically significant increase in normalized mRNA expression of Pla2g4a was detected in LPS-treated APdE9 mice compared to saline-treated APdE9 and LPS-treated WT mice (*p* = 0.01). After that, we investigated the changes in absolute protein expression of cPLA2α in the brain cortical tissue of the investigated mouse models using LC-MS/MS-based QTAP analysis (Figure 1B). The study revealed a significant increase in cPLA2α protein expression in LPS-treated APdE9 mice compared to saline-treated WT mice (*p* = 0.01). Similarly, in terms of cPLA2 activity in the brain cortical tissue of the exposed mice, a statistically significant increase (*p* = 0.01) was observed in LPS-treated APdE9 mice compared to saline-treated WT mice (Figure 1C).

### 3.2. NFκB Signaling

**Figure 2:**
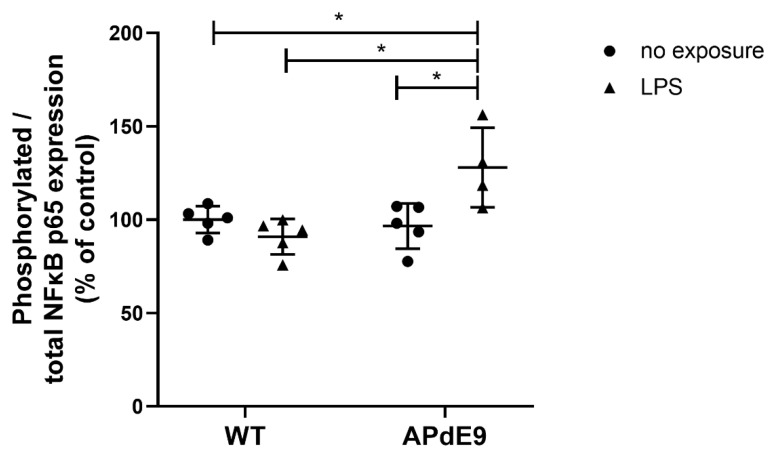
Phosphorylated/total NFκB p65 expression in the cerebral cortical tissue in saline-treated female wild-type (WT) control mice vs. female WT mice treated with LPS, and female APdE9 mice treated with either saline or LPS. The line represents group mean and whiskers—the SD (*n* = 4–5 per group). The statistical significance of changes in gene expression between the groups was analyzed by a Kruskal–Wallis test followed by a Dunn’s multiple comparisons post-hoc test. Statistically significant differences are marked with asterisks, where * indicates *p* < 0.05.

To investigate NFκB signaling, we measured phosphorylated to total NFκB p65 expression in the brain cortical tissue of the investigated mouse models. We observed a significant difference between the study groups (*p* = 0.01) after Kruskal–Wallis analysis. The Dunn’s multiple comparisons post-hoc test revealed significantly higher phosphorylated to total NFκB p65 expression in the brain cortices of LPS-treated APdE9 mice compared to all three other groups (Figure 2).

### 3.3. Inflammatory Responses and Oxidative State in the Brain Cortices of LPS-Treated Mice

**Figure 3:**
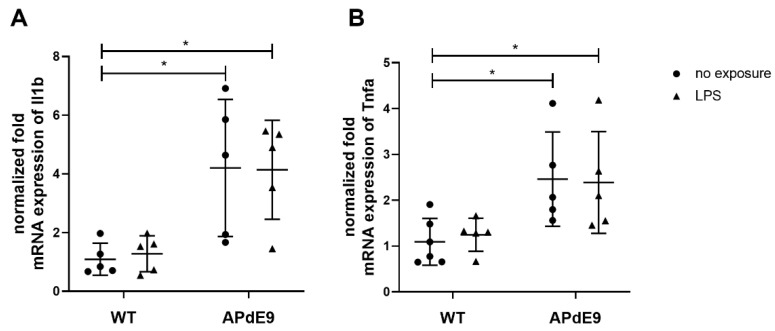
Comparison of normalized fold mRNA expression of pro-inflammatory cytokines in-terleukin-1 beta (Il1b) (**A**) and tumor necrosis factor alpha (Tnfa) (**B**) in the cerebral cortical tissue in saline-treated female wild-type (WT) control mice vs. female WT mice treated with LPS, and female APdE9 mice treated with either saline or LPS. The line represents group mean and whiskers—the SD (*n* = 4–6 per group). The statistical significance of changes in gene expression between the groups was analyzed by a Kruskal–Wallis test followed by a Dunn’s multiple comparisons post-hoc test. Statistically significant differences are marked with asterisks, where * indicates *p* < 0.05.

**Figure 4:**
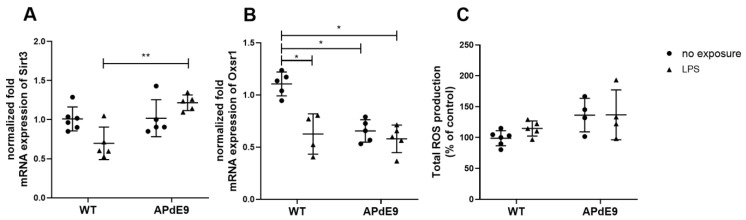
Comparison of normalized fold mRNA expression of oxidative stress markers sirtuin-3 (Sirt3) (**A**) and oxidative stress responsive kinase 1 (Oxsr1) (**B**) and the production of reactive oxygen species (ROS) expressed as % to control (**C**) in the cerebral cortical tissue in saline-treated female wild-type (WT) control mice vs. female WT mice treated with LPS, and female APdE9 mice treated either with saline or LPS. The line represents group mean and whiskers—the SD (*n* = 4–6 per group). The statistical significance of changes in gene expression between the groups was analyzed by a Kruskal–Wallis test followed by a Dunn’s multiple comparisons post-hoc test. Statistically significant differences are marked with asterisks, where * indicates *p* < 0.05, ** *p* < 0.01.

The changes in gene expression of pro-inflammatory cytokines, Il1b and Tnfa, and oxidative stress markers, Sirt1 and Oxsr1, were assessed in the brain cortices of the investigated mouse models using qRT-PCR (Figure 3 and Figure 4). Kruskal–Wallis analysis revealed statistically significant differences in normalized fold mRNA expression of both pro-inflammatory cytokines, Il1b (*p* = 0.01) and Tnfa (*p* = 0.01) (Figure 3A,B). The Dunn’s multiple comparisons post-hoc test revealed a statistically significant increase in normalized mRNA expression on Il1b and Tnfa in saline- and LPS-treated APdE9 mice compared to saline-treated WT mice.

While comparing the gene expression of Sirt3 and Oxsr1 between the study groups using Kruskal–Wallis analysis, we detected a significant difference between the study groups (*p* = 0.01) for both genes (Figure 4A,B). The applied Dunn’s multiple comparisons post-hoc test used for comparison of Sirt3 gene expression between the treatment groups revealed a statistically significant increase in Sirt3 normalized fold mRNA expression in LPS-treated APdE9 mice compared to WT mice treated with LPS (*p* = 0.009) (Figure 4A). Moreover, significantly decreased Oxsr1 normalized fold mRNA expression was observed in all of the treatment groups compared to saline-treated WT mice (Figure 4B). We compared the production of ROS in the brain cortical tissue of the studied mouse models. Kruskal–Wallis analysis did not reveal a statistically significant differences between the study groups (Figure 4C).

## 4. Discussion

The role of cPLA2 in mediating inflammatory and oxidative responses in microglia, neurons, and astrocytes has been previously demonstrated [4,11,36]. Multiple studies have shown that cPLA2 gene and protein expression, as well as levels of the phosphorylated form were elevated in AD brains compared to healthy ones [13,17,37]. Moreover, increased cPLA2 activity was found in the hippocampus, but not in the cortex of hAPP transgenic mice [17]. In this AD model, genetic ablation of the enzyme provided protective effects against Aβ-mediated behavioral, learning and memory impairment [17]. Moreover, cPLA2-knock-out microglia possessed decreased oxidative stress and inflammatory status in response to LPS treatment [11]. 

In the present study, we investigated for the first time the impact of systemic chronic LPS treatment on the expression and activity of brain cortical cPLA2 in a transgenic AD model, i.e., APdE9 mice. Our study revealed the significant effect of LPS treatment on the elevated protein expression and activity of cPLA2 in the APdE9 mouse cortex as compared to saline-treated WT mice. These data support our previous findings of altered lipid metabolism in the same animals as used in the current study [24]. Thus, the number of significantly elevated LPCs and decreased PCs were higher in the brain cortex of APdE9 mice treated with LPS as compared to saline-treated WT and APdE9 mice as well as LPS-treated WT mice [24]. As cPLA2 mediates the formation of LPCs from PCs, the observed changes in lipid metabolism in these models can be explained by genotype-specific and LPS-induced effects on the protein expression and activity of cPLA2 in the brain cortical tissue of APdE9 mice. Interestingly, the increased activity of cPLA2 did not result in elevated brain cortical levels of arachidonic acid, the levels of which did not differ between the study groups [24]. These findings can be explained by the fact that the impact of elevated cPLA2 activity on arachidonic acid levels is limited by other factors. These factors include incorporation of free arachidonic acid into membrane phospholipids through esterification by acyl-CoA synthetases (ACSLs) and the transfer of arachidonic acid from arachidonyl-CoA to lysophospholipids by various acyl-CoA lysophospholipids acyltransferases (LPATs) [38,39]. Thus, both ACLS4- and LPATs-mediated membrane incorporation processes could reduce the levels of free arachidonic acid in neuronal and glial cells independently of increased activity of cPLA2.

The expression of cPLA2α is controlled at the transcriptional level and by posttranslational modifications. At the transcriptional level, cPLA2α expression is induced through several signaling pathways involving NF-κB [40]. In the present study, gene expression of Pla2g4a was increased due to the effects of both genotype and LPS treatment. We observed similar changes in the activation of NF-κB signaling in the brain cortical tissue of the investigated mice. These findings provide evidence of the NF-κB-mediated induction of Pla2g4a expression due to LPS-induced systemic inflammation in APdE9 mice. Pro-inflammatory cytokines, such as IL-1β and TNF-α, demonstrated an inducing effect on the gene expression of human cPLA2α [40,41]. Multiple administrations of LPS in 3 × Tg-AD mice have been shown to lead to elevated brain gene expression and protein levels of IL-1β and TNF-α [42]. In the present study, increased gene expression of Il1b and Tnfa resulted from the AD genotype, but not from the chronic LPS treatment indicating that pro-inflammatory cytokines do not contribute to the elevated expression and activity of cPLA2 in LPS-exposed APdE9 mice.

Cellular activation of cPLA2 occurs rapidly by post-translational processes, such as increases in intracellular calcium concentrations and phosphorylation by MAPK through multiple receptor-mediated pathways in response to cell stimulation. Thus, cell stimulation occurs after activation of a ligand for a receptor, such as adenosine triphosphate (ATP) or platelet-derived growth factor (PDGF), phospholipase C (PLC) via either a G protein-dependent or -independent process [6]. This results in the production of intracellular messengers DAGs and IP3. The increase in their concentrations led to the activation of protein kinase C (PKC) and intracellular calcium mobilization [6]. A rise in calcium concentration results in translocation of cPLA2 from the cytosol to the cellular membrane, where its substrates, phospholipids, are localized [6]. This is a crucial step for cPLA2 activation, which plays an important role in the partial activation of the enzyme even in the absence of phosphorylation. In our previous study, we revealed that LPS-induced systemic inflammation caused a significant rise in various DAGs in the brain cortical tissue of APdE9 mice [24]. Thus, the elevated production of DAGs could be one of the reasons for the LPS-induced activation of cPLA2 in the brain cortical tissue of APdE9 mice observed in the present study. In addition, cPLA2 activity depends on MAPK-mediated phosphorylation [5,6]. However, the effect of LPS-induced systemic inflammation on cPLA2 phosphorylation in APdE9 mice has not been investigated in the present study. This limits our knowledge to make a conclusion about the impact of cPLA2 phosphorylation on the elevated activity of the enzyme due to LPS treatment in the mice investigated in this study. Future research should focus on this issue.

Shelat et al. (2008) demonstrated that Aβ_1–42_ induce ROS production from cortical neurons via NADPH oxidase activation [43]. The produced ROS caused activation of extracellular signal-regulated kinase 1/2 (ERK 1/2), phosphorylation of cPLA2α, and the release of arachidonic acid [43]. Moreover, Chuang et al. (2015) revealed that cPLA2 plays an important role in microglial activation [11]. Thus, LPS-induced ROS production was inhibited by a specific cPLA2 inhibitor, thus suggesting an association between cPLA2 and LPS-induced oxidative stress in murine immortalized BV-2 microglial cells [11]. Therefore, in the present study we investigated the oxidative status in the brain cortical tissue by quantifying the gene expression of oxidative stress markers, Sirt3 and Oxcr1, as well as the production of ROS. Interestingly, we did not observe any effect on ROS production and Sirt3 due to LPS treatment, while both genotype and LPS treatment affected the gene expression of Oxsr1. However, the changes in the gene expression of Oxsr1 did not show an association with the elevated cPLA2 activity observed in APdE9 mice treated with LPS. These findings demonstrate that LPS-mediated changes in the expression and activity of cPLA2 in APdE9 mice were not associated with the changes in oxidative status in the brain of these models. 

## 5. Conclusions

In conclusion, the present study revealed that chronic LPS treatment resulted in genotype-specific increases in the protein expression and activity of cPLA2. Together with findings from our previous study, these data indicate that LPS-triggered cPLA2 activation in APdE9 mice may be caused by increased transcription of Pla2g4a associated with NF-κB signaling as well as facilitated translocation to cell membranes due to a rise in the formation of DAGs. Moreover, there is evidence that LPS-induced activation of cPLA2 leads to altered formation of brain cortical LPCs from PCs in APdE9 mice. However, it does not affect arachidonic acid levels or facilitate oxidative stress in the brain cortex of this AD model. All in all, the present study provides novel information about the impact of systemic inflammation on cPLA2 expression and activity in AD. The findings of this study suggest that inhibition of cPLA2 may be a promising strategy for the treatment and prevention of AD accompanied by chronic or repeated peripheral infections and inflammation. 

## Figures and Tables

**Table 1:** Primer sequences for SYBR Green qRT-PCR.

Gene	Forward Primer	Reverse Primer
Pla2g4a	5′-AGAAGACCTGGGAAGTGTGAGA-3′	5′-TCTGGAGTGTCCAGCATATCG-3′
Sirt3	5′-GCTACATGCACGGTCTGTCGAA-3′	5′-CAATGTCGGGTTTCACAACGCC-3′
Oxsr1	5′-ACGAGCCAACCATTGCTACA-3′	5′-ACTGACGCCGAAATCTGCAA-3′
Tnfa	5′-GTACCTTGTCTACTCCCAGGTTCTCT-3	5′-GTGTGGGTGAGGAGCACGTA-3′
Il1b	5′-GCAACTGTTCCTGAACTCAACT-3′	5′-ATCTTTTGGGGTCCGTCAACT-3′
Actb	5′-AAGTCCCTCACCCTCCCAAAAG-3′	5′-ACACAGAAGCAATGCTGTCACC-3′

## Data Availability

The data presented in this study are available upon reasonable request from the corresponding author.
